# Molecular characterization of *Cryptosporidium* in ruminants and observation of natural infection by *Cryptosporidium andersoni* in sheep from Paraná, Brazil

**DOI:** 10.1590/S1984-29612023076

**Published:** 2023-12-04

**Authors:** Luciane Holsback, Ellen de Souza Marquez, Marcelo Alves da Silva, Petrônio Pinheiro Porto, João Luis Garcia, Felippe Danyel Cardoso Martins, Mércia de Seixas

**Affiliations:** 1 Setor de Veterinária e Produção Animal, Universidade Estadual do Norte do Paraná, Bandeirantes, PR, Brasil; 2 Departamento de Medicina Veterinária Preventiva, Universidade Estadual de Londrina, Londrina, PR, Brasil

**Keywords:** Cryptosporidiosis, bovine, sheep, genotyping, RFLP, genetic sequencing, Criptosporidiose, bovino, ovino, genotipagem, RFLP, sequenciamento genético

## Abstract

The aim of this study was to identify *Cryptosporidium* species found in cattle and sheep in Paraná, southern region of Brazil. Individual fecal samples from 458 bovines and 101 sheep were submitted for molecular analysis by PCR and nested PCR using specific primers for sequences of the 18S ribosomal unit (rRNA). Positive samples were analyzed using restriction fragment length polymorphism (RFLP), followed by genetic sequencing for species confirmation. The occurrence of *Cryptosporidium* was 11.27% (63/559). The highest occurrence was detected in lambs (12/59, 20.33%). From the 63 positive samples, it was possible to identify the species in 58 of them by RFLP and genetic sequencing. Five species of *Cryptosporidium* were identified: *Cryptosporidium andersoni, Cryptosporidium bovis, Cryptosporidium ryanae*, *Cryptosporidium xiaoi,* and *Cryptosporidium parvum*. The most prevalent species was *C. andersoni* (41.38%) and the least predominant was *C. parvum* (10.34%). The most abundant species of *Cryptosporidium* in dairy calves were *C. andersoni* (11/25) and *C. ryanae* (6/25). Of the 17 positive sheep, nine (52.94%) were infected with *C. andersoni*. This finding is the first report on the occurrence of *C. andersoni* in naturally infected sheep in Brazil and the first observation of a high absolute occurrence of this *Cryptosporidium* species in sheep.

## Resumo

O objetivo deste estudo foi identificar espécies de *Cryptosporidium* em bovinos e ovinos do Paraná, região sul do Brasil. Amostras de fezes de 458 bovinos e 101 ovinos foram individualmente submetidas à análise molecular por PCR e *nested* PCR, utilizando-se iniciadores específicos para sequências da unidade ribossomal 18S (rRNA). As amostras positivas foram analisadas pelo polimorfismo de comprimento de fragmento de restrição (RFLP), seguido de sequenciamento genético para confirmação da espécie. A ocorrência de *Cryptosporidium* foi de 11,27% (63/559). Observou-se maior ocorrência em cordeiros (20,33%). Das 63 amostras positivas, foi possível identificar as espécies em 58 amostras por RFLP e sequenciamento genético. Foram identificadas cinco espécies de *Cryptosporidium: Cryptosporidium andersoni*, *Cryptosporidium bovis*, *Cryptosporidium ryanae*, *Cryptosporidium xiaoi* e *Cryptosporidium parvum*. A espécie mais predominantemente encontrada foi *C. andersoni* (41,38%) e a menos foi *C. parvum* (10,34%). As espécies mais abundantes de *Cryptosporidium,* em bezerros leiteiros, foram *C. andersoni* (11/25) e *C. ryanae* (6/25). Dos 17 ovinos positivos, nove (52,94%) estavam infectados com *C. andersoni*. Este achado é o primeiro relato sobre a ocorrência de *C. andersoni* em ovinos naturalmente infectados no Brasil e a primeira observação de alta ocorrência absoluta desta espécie de *Cryptosporidium* em ovinos.

## Introduction

*Cryptosporidium* spp. are protozoa belonging to the phylum Apicomplexa known to affect the gastrointestinal tract of animals, including humans. Transmission of *Cryptosporidium* occurs mainly through ingestion of fecally contaminated water or food or by direct contact with infected animals (zoonotic), people (anthroponotic), or contaminated surfaces (Meisel et al., 1976; Huang & White, 2006).

Until 1970, cryptosporidiosis was considered a rare and opportunistic infection (Xiao et al., 2004). However, following reports of infection in cattle and humans, cryptosporidiosis came to the attention of researchers for its anthropozoonotic potential and for causing clinical and subclinical disease in animals and humans. To date, a total of 44 *Cryptosporidium* and *Cryptosporidium*-like species have been described from animals and humans (Feng et al., 2018).

The identification of *Cryptosporidium* genotypes in ruminants in the state of Paraná was performed by Toledo et al. (2017), Snak et al. (2017) and Oliveira et al. (2021) and in captive birds by Nakamura et al. (2009). Epidemiological studies of parasitic diseases in the Northern Pioneer mesoregion are scarce. However, high parasite loads in ruminants are reported, which may reflect poor sanitary conditions and management in this region (Holsback et al., 2016). Due to the lack of knowledge about potentially zoonotic species in the region, and the importance of this for one health, the aim of this investigation was to identify *Cryptosporidium* species from different age categories of cattle and sheep in the north of Paraná.

## Material and Methods

**Figure 1:**
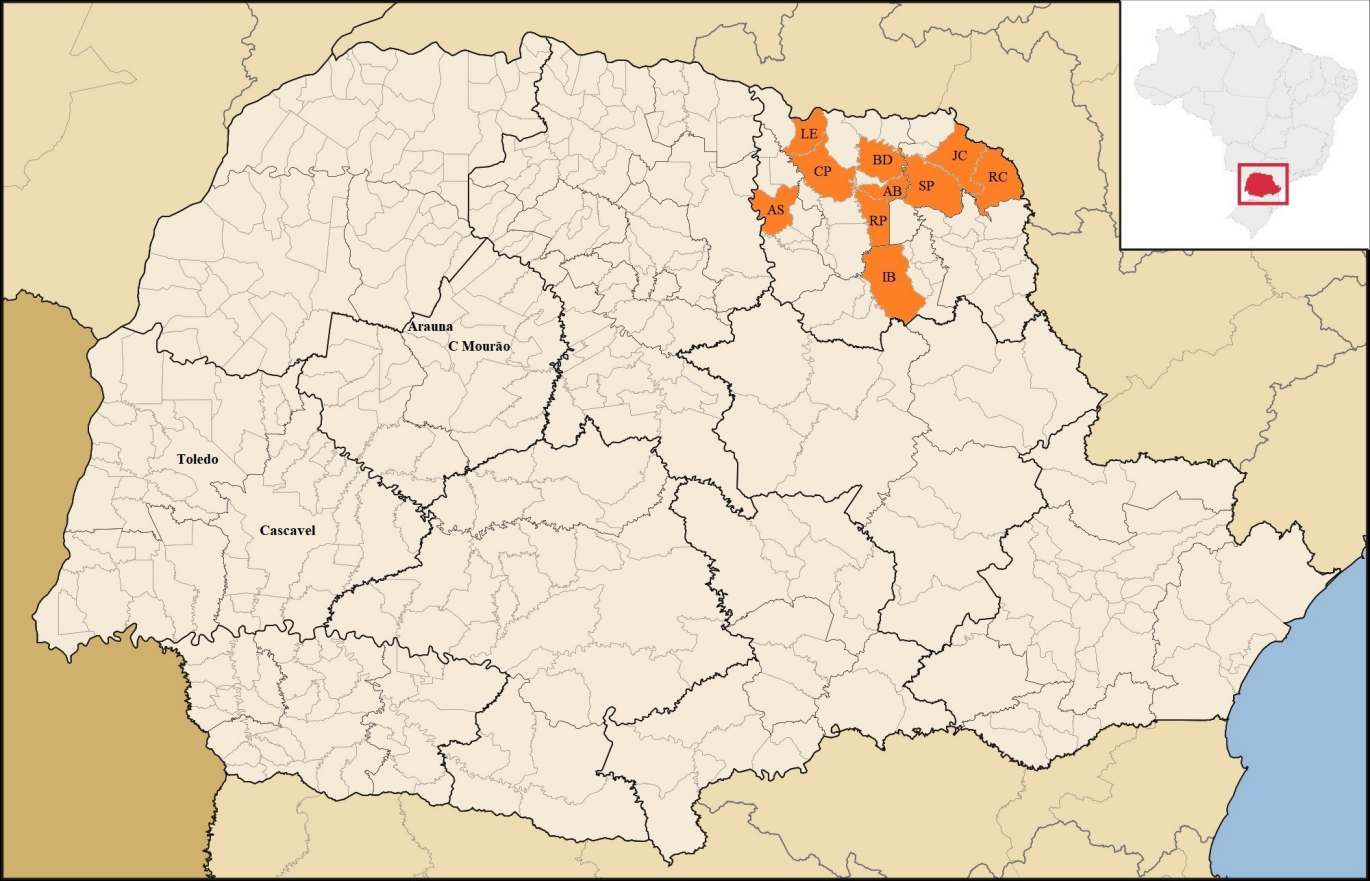
Map of the State of Paraná showing the municipalities (area filled/orange) of the present study in the Northern Pioneer mesoregion, and the municipalities of Arauna, Campo Mourão (C. Mourão), and Cascavel where previous studies were carried out in the same state. Upper right map: Map of Brazil showing the State of Paraná (square/red). AS: Assaí; LE: Leópolis; CP: Cornélio Procópio; RP: Ribeirão do Pinhal; IB: Ibaiti; AB: Abatiá; BD: Bandeirantes; SP: Santo Antônio da Platina; JC: Jacarezinho; RC: Ribeirão Claro. Source: List of mesoregions and microregions of Paraná (Wikipédia, 2022).

Fecal samples from 458 bovines (12 dairy cows older than 36 months; 37 beef cows older than 24 months; 294 post-weaned dairy calves from six to 12 months; and 115 pre-weaned beef calves from four to six months), and 101 sheep (42 ewes more than 18 months, and 59 post-weaned lambs from four to seven months) were collected from 44 properties in the municipalities of Abatiá (n = 6), Assaí (n = 75), Bandeirantes (n = 25), Cornélio Procópio (n = 18), Ibaiti (n = 44), Jacarezinho (n = 34), Leópolis (n = 152), Ribeirão Claro (n = 20), Ribeirão do Pinhal (n = 74), and Santo Antônio da Platina (n = 111), located in the Northern Pioneer mesoregion of the State of Paraná (Figure 1). All animals were healthy during the sampling.

All the samples were subjected to DNA extraction using a commercial kit (NucleoSpin Tissue, Macherey-Nagel, DuÈren, Germany). To detect *Cryptosporidium* spp., fragments of the 18S rRNA gene were amplified using a nested PCR (nPCR) assay (Xiao et al., 1999). Samples were processed in triplicate, and each reaction mixture contained 1x PCR Buffer, 200 μM dNTP, 2.5 mM MgCl_2_, 400 nM each primer, 1.25 U of Platinum Taq DNA Polymerase, 2 μL of the extracted DNA from each sample, and ultrapure water. The material obtained in the first reaction (25 µL) was diluted with 50 µL of ultrapure water, and 2 µL of this previously diluted amplicon was then used for the second reaction. Amplification conditions for both the first and second reactions were as follows: five min at 95°C; followed by 35 cycles of 45 s at 94°C, 45 s at 55°C, and 60 s at 72°C; with the final extension step, five min at 72°C. Obtained PCR products were subjected to electrophoresis in a 1.5% agarose gel (UltraPure™ Agarose, Invitrogen, Waltham, MA, USA) stained with DNA gel stain (SYBR™ Safe, Invitrogen, Waltham, MA, USA) and visualized on ultraviolet light.

Second-round PCR products positive for *Cryptosporidium* spp. were subjected to restriction fragment length polymorphism (RFLP) aiming to characterize the *Cryptosporidium* species; the DNA was digested with restriction enzymes *Ssp*I, *Ase*I, *MboII*, and *DdeI* (Xiao et al., 1999; Feng et al., 2007). The reaction was performed with 5 μL of DNA, 2 μL of a specific 10X restriction buffer, 3 U of enzyme (New England Biolabs, Ipswich, MA, USA), and ultrapure water up to a 20 μL of final reaction volume. Digestion was performed at 37°C for one hour, and the products were subjected to electrophoresis in a 2.5% agarose gel stained with SYBR™ Safe.

Selected PCR products for the SSU-rRNA gene were sequenced in both directions with the forward and reverse primers used in the secondary PCRs. Sequencing was performed using a BigDye™ Terminator v3.1 Cycle Sequencing Kit (Applied Biosystems, USA) and an ABI3500 sequencer genetic analyzer (Applied Biosystems, Life Technologies™, Carlsbad, CA, USA). The resulting nucleotide sequences were compared with the standard *Cryptosporidium* sequences in GenBank using the Basic Local Alignment and Search Tool (BLAST) and by manual alignment in BioEdit software (Biological Sequence Alignment Editor).

Variables such as species, age, and animal category (dairy cattle and beef cattle) were analyzed for association with the presence/absence of *Cryptosporidium* DNA in feces. The comparison between the *Cryptosporidium* frequency data and the epidemiological variables was performed using the chi-squared test or Fisher's exact test. The magnitude of the association was determined by Odds Ratio (OR). The analysis was performed in GraphPad Prism v. 6.01 (GraphPad Software, San Diego, EUA), with the level of statistical significance at 5%.

## Results and Discussion

The occurrence of *Cryptosporidium* infection was 11.27% (63/559), with lambs showing a higher prevalence (p=0.0271, OR=2.292) (20.34%) than calves (10.02%). The prevalence of *Cryptosporidium* infection in cows was 10.2% (5/49) and in sheep, 11.9% (5/42). The parasite was found on 38.64% (17/44) of the farms.

Of the 63 PCR-positive samples, five samples showed fainted bands and could not be correctly sequenced. The samples showed similarity of 99.02 to 99.87% to the *Cryptosporidium* species, being 24 (41.38%) with *C. andersoni* (GenBank accession number MT648437.1), 13 (22.41%) with *C. ryanae* (MF671876.1), 8 (13.79%) with *C. bovis* (OP861764.1), 7 (12.07%) with *C. xiaoi* (KP004203.1), and 6 (10.34%) with *C. parvum* (MH754179.1).

The GenBank nucleotide sequence accession numbers for the partial sequences generated in the present study are: *C. bovis* (OR737845, OR738302, and OR736735), *C. xiaoi* (OR737885, and OR738637), *C. ryanae* (OR743625), *C. andersoni* (OR743623, and OR738639), and *C. parvum* (OR743929).

The present study found that the prevalence of *Cryptosporidium* was not different among cattle and sheep of different ages, suggesting that adult ruminants might act as reservoirs for *Cryptosporidium*. In this survey, six animals were diagnosed with *C. parvum*; three were from dairy calves (two from the same property), one sample was from a beef calf, and the two remaining originated from a beef cow and a sheep, all from different properties.

*Cryptosporidium parvum* is the most prevalent species in young calves in the pre-weaning phase (<2 months old) and shows low host specificity with some genotypes considered of high zoonotic potential (Feng et al., 2007). The animals examined in this research were older and all asymptomatic; however, the symptoms of cryptosporidiosis in cattle are dependent on the infecting species and immune status of the host (Coklin et al., 2009).

Of the 24 *C. andersoni* samples found, 11 (45.83%) were from post-weaned dairy calves and 5 (20.83%) from lambs (Table 1). According to Paz e Silva et al. (2013), the prevalence of *C. andersoni* infection in calves in the pre-weaning phase is relatively low, with the highest infection rate observed mainly in post-weaned calves. However, in this research, we did not analyze pre-weaned dairy calves, so it was not possible to compare the infection rates of older calves.

**Table 1:** Distribution of *Cryptosporidium* species in post-weaned dairy calf, pre-weaned beef calf, dairy cow, beef cow, post-weaned lamb, and ewe and total positive samples with the relative frequency (RF) per species.

		Positive animals/RF(%)					
		Post weaned Dairy calf	Pre-weaned Beef calf	Dairy cow	Beef cow	Post weaned lamb	Ewe
Species of *Cryptosporidium* found	Total (RF%)	25 (43.10%)	12 (20.69%)	1 (1.72%)	3 (5.17%)	12 (20.69%)	5 (8.62%)
*C. andersoni*	24 (41.38%)	11 (45.83%)	2 (8.33%)	1 (4.17%)	1 (4.17%)	5 (20.83%)	4 (16.67%)
*C. bovis*	8 (13.79%)	5 (62.50%)	3 (37.50%)	0	0	0	0
*C. parvum*	6 (10.34%)	3 (50%)	1 (16.67%)	0	1 (16.67%)	0	1 (16.67%)
*C. ryanae*	13 (22.41%)	6 (46.15%)	6 (46.15%)	0	1 (7.69%)	0	0
*C. xiaoi*	7 (12.07%)	0	0	0	0	7 (100%)	0

**Figure 2:**
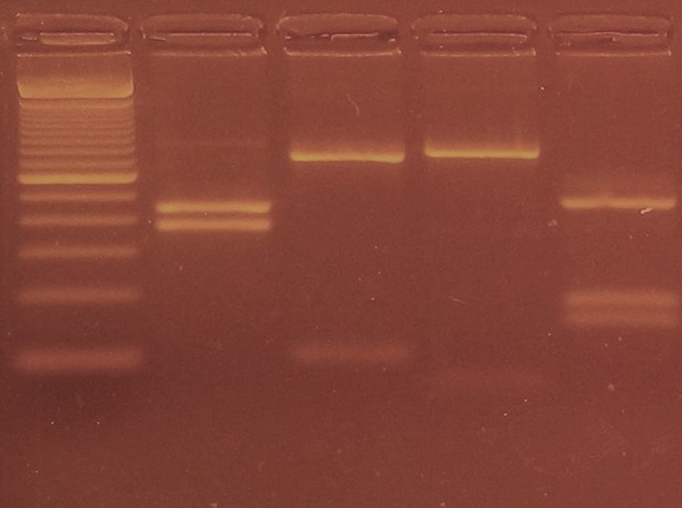
Differentiation of *Cryptosporidium andersoni* by RFLP analysis from post-weaned lamb sample. Secondary PCR products were digested with restriction enzymes SspI, VspI, AseI, and DdeI, and the results of the digestions are shown. Lane 1 (Marker) represents 100bp DNA size marker; Lanes 2, 3, 4, and 5 represent the results of the digestions of restriction enzymes SspI (384 and 448bp), AseI (102 and 730bp), MboII (63 and 769bp), and DdeI (156, 186 and 470bp) respectively.

Of the 17 *Cryptosporidium* spp. positive sheep, nine (53%) were characterized as *C. andersoni* (Table 1, Figure 2). These animals came from different properties in the cities of Santo Antônio da Platina, Ibaiti, Ribeirão do Pinhal and Leópolis (Table 2). All the sheep sampled share pastures with cattle at some period during the year. Also, owners reported rotating grazing between cattle and sheep intending to reduce the parasitic load of ticks in pastures, which could explain the high prevalence of *C. andersoni* in sheep.

**Table 2:** Total number of post-weaned dairy calves, pre-weaned beef calves, dairy cows, beef cows, post-weaned lambs and ewes evaluated by municipality and number of animals positive for PCR, relative frequency (%) and species of *Cryptosporidium* found.

Municipalities	Total	Positive PCR/ Relative frequency(%)	Species of ***Cryptosporidium***
**ASSAÍ**			
Number of properties	2		
Post weaned Dairy calf	28	6 (21.4%)	*C. bovis, C. andersoni, C. ryanae*
Pre-weaned Beef calf	25	4 (16.0%)	*C. parvum, C. ryanae*
Dairy cow	6	0	~
Beef cow	7	1 (14.3%)	*was not possible to identify the specie*
Post weaned lamb	9	3 (33.3%)	*C. xiaoi*
**SANTO ANTÔNIO DA PLATINA**			
Number of properties	7		
Post weaned Dairy calf	57	1 (1.8%)	*C. ryanae*
Pre-weaned Beef calf	16	3 (18.8%)	*C. bovis*
Beef cow	9	0	*~*
Post weaned lamb	18	2 (11.1%)	*C. andersoni*
Ewe	11	0	~
**IBAITI**			
Number of properties	2		
Post weaned Dairy calf	15	7 (46.7%)	*C. bovis, C. andersoni, C. ryanae*
Pre-weaned Beef calf	9	3 (33.3%)	*C. ryanae, C. andersoni*
Dairy cow	6	1 (16.7%)	*C. andersoni*
Beef cow	6	2 (33.3%)	*C. ryanae, C. andersoni*
Ewe	8	3 (37.5%)	*C. andersoni*
**RIBEIRÃO DO PINHAL**			
Number of properties	3		
Post weaned Dairy calf	26	0	~
Pre-weaned Beef calf	23	2 (8.7%)	*C. bovis, C. andersoni*
Beef cow	7	1 (14.3%)	*C. parvum*
Post weaned lamb	7	0	~
Ewe	11	2 (18.2%)	*C. andersoni, C. parvum*
**RIBEIRÃO CLARO**			
Number of properties	1		
Pre-weaned Beef calf	17	0	~
Beef cow	2	0	~
Post weaned lamb	1	0	~
**JACAREZINHO**			
Number of properties	7		
Post weaned Dairy calf	34	4 (11.8%)	*C. andersoni, C. ryanae*
**BANDEIRANTES**			
Number of properties	3		
Post weaned Dairy calf	25	0	~
**CORNÉLIO PROCÓPIO**			
Number of properties	3		
Post weaned Dairy calf	18	2 (11.1%)	
**LEÓPOLIS**			
Number of properties	15		
Post weaned Dairy calf	85	7 (8.2%)	*C. andersoni, C. parvum, C. ryanae*
Pre-weaned Beef calf	25	2 (8.0%)	*C. ryanae*
Beef cow	6	0	*~*
Post weaned lamb	24	7 (29.2%)	*C. xiaoi, C. andersoni*
Ewe	12	0	*~*
**ABATIÁ**			
Number of properties	1		
Post weaned Dairy calf	6	0	~

Dalimi & Tahvildar (2017) found a high relative occurrence (20/22) of *C. andersoni* in sheep in Iran, however, the absolute occurrence of *C. andersoni* in the 1,300 sheep analyzed was 1.54%. In this study, of 101 sheep fecal samples analyzed, we found *C. andersoni* in nine (8.91%).

In a study about the prevalence of *Cryptosporidium* in sheep globally including molecular data via meta-analysis concluded that *C. parvum* is the dominant species in Europe while *C. xiaoi* is the dominant species in Oceania, Asia, and Africa (Chen et al., 2022). Fiuza et al. (2011) and Paz e Silva et al. (2014) have found several species of *Cryptosporidium* in sheep, including C. *ubiquitum* which is considered dominant in South America (Chen et al., 2022). In our study, we did not identify *C*. *ubiquitum* in sheep.

*Cryptosporidium andersoni* infection in sheep has not been reported yet in Brazil. This species of *Cryptosporidium* is known to infect bovine abomasum (Lindsay et al., 2000), marmot, camel, and bison (Ryan et al., 2005). Kvác et al. (2004) performed an experimental infection with *C. andersoni* in four lambs aged four months. None of the animals showed clinical or pathological aspects of cryptosporidiosis in the autopsy. No visible changes were detected in the abomasum or other examined organs and histological examination proved negative for *Cryptosporidium*. These authors concluded that the isolate used was noninfective for lambs at four months old. Models for the experimental infection should produce levels of infection and disease in high prevalence. Furthermore, the pathogenesis of disease that occurs after artificial infection must mimic disease patterns that occur naturally. The low number of infected animals and the health status may have contributed to the infection's failure.

Ryan et al. (2005), Quílez et al. (2008), Wang et al. (2010), Koinari et al. (2014), and Yang et al. (2014) described a low occurrence of *C. andersoni* in sheep which may contribute to the perception that *C. andersoni* is not able to infect such species, but our data provide evidence to support that *C. andersoni* infects sheep.

Considering the zoonotic potential of *C. andersoni* cited in several reports and the findings in sheep in our study, it is important to consider the sheep as a source of *C. andersoni* infection for humans and other animals in Brazil, although the extent of its zoonotic transmission needs to be precisely determined (Ryan et al., 2021). Studies on the genetic diversity of *Cryptosporidium* in sheep should be explored and deserve further investigation in Brazil.

## Conclusion

It was concluded that ruminants in this region are infected with a wide variety of *Cryptosporidium* species. It is essential to consider sheep as hosts and sources of infection of *C. andersoni.* Consequently, to stimulate the scientific community to study this parasite in this animal species and in humans is essential, especially in Brazil where there are no reports in humans.

To our knowledge, this is the first report of *C. andersoni* occurrence in naturally infected sheep in Brazil and the first report of *C. andersoni* high absolute occurrence in the ovine host.
